# Why Do Japanese People Use Masks Against COVID-19, Even Though Masks Are Unlikely to Offer Protection From Infection?

**DOI:** 10.3389/fpsyg.2020.01918

**Published:** 2020-08-04

**Authors:** Kazuya Nakayachi, Taku Ozaki, Yukihide Shibata, Ryosuke Yokoi

**Affiliations:** ^1^ Faculty of Psychology, Doshisha University, Kyotanabe, Japan; ^2^ Graduate School of Psychology, Doshisha University, Kyotanabe, Japan

**Keywords:** coronavirus infection, risk reduction, risk perceptions, mask, societal norms, affect heuristic, 2019 coronavirus

## Abstract

Wearing masks against 2019 coronavirus (COVID-19) is beneficial in suppressing pandemic spread, not through preventing the wearer from being infected but by preventing the wearer from infecting others. Despite not providing much protection, the custom of wearing masks has prevailed in East Asia from the early stages of the pandemic, especially in Japan, to such an extent that it caused a shortfall in supply. Why do many Japanese people wear masks during the COVID-19 pandemic, even though masks are unlikely to prevent them from getting infected? We examined six possible psychological reasons for wearing masks: three involved expectations about the risk of infection and three involved other driving psychological forces. The results of our nationwide survey revealed that people conformed to societal norms in wearing masks and felt relief from anxiety when wearing masks. However, risk reduction expectations did not affect mask usage. The social psychological motivations successfully explained much about mask usage. Our findings suggest that policymakers responsible for public health should consider social motivations when implementing public strategies to combat the COVID-19 pandemic.

## Introduction

Why do many Japanese people wear masks during the 2019 coronavirus (COVID-19) pandemic, even though masks are unlikely to prevent them from getting infected? Wearing masks against COVID-19 is beneficial in suppressing pandemic spread, not through preventing the wearer from being infected but by preventing the wearer from infecting others, according to suggestions from the World Health Organization (WHO, 2020a,b,c) and lessons from previous pandemics, such as the 2003 severe acute respiratory syndrome (SARS) pandemic and the 2009 influenza A virus subtype H1N1 pandemic (Mniszewski et al., 2014; Leung et al., 2020). The Director-General of the Chinese Center for Disease Control and Prevention also stated that, “not wearing masks to protect against coronavirus is a “big mistake”” in terms of preventing the spread of infection, but not in terms of personal infection prevention (Cohen, 2020). Despite not providing much protection, the custom of wearing masks has prevailed in East Asia from the early stages of the pandemic, especially in Japan (Yamagata et al., 2020); to such an extent that it caused a shortfall in supply (BBC, 2020). What are the psychological reasons prompting an individual to take a measure from which they cannot directly benefit? Individuals’ cumulative actions are beneficial to society, but not directly beneficial to themselves. In our survey, we examined six possible psychological reasons for wearing masks: three involved individuals’ perception of the severity of the disease and the efficacy of masks in reducing the infection risks both for themselves and for others; the remaining three involved other psychological driving forces.

The altruistic intention could be the primary reason for wearing masks, to avoid spreading the disease to others. Although perfect altruism seems impossible, people often behave to benefit others at a certain cost to themselves (Batson et al., 1981; Schwartz and Howard, 1981). Altruistic risk reduction to others is favorable for the whole of society; however, does such an altruistic motivation work well during a dreadful pandemic? Another motivation to reduce risk is self-interest that is, protecting oneself against the virus, even if this is a misperception. If people are confident that masks will protect them against infection, they are likely to wear them. Perceived seriousness of the disease could be another reason to wear a mask. The more an individual sees the disease as serious, the higher is the person’s motivation to take action. Theories of protection behavior such as the protection motivation theory (Rogers, 1975, 1983) and the protective action decision model (Lindell and Perry, 1992, 2012) posit that people cope well with risks when they perceive a threat as serious, and take action when they perceive the action as effective in mitigating associated damage.

Those three reasons are predicated on reducing the risk of infection to others or to oneself. However, people’s actions are not necessarily connected to the original motivating purpose of the action. Three factors could result in collective mask-wearing even in the absence of an intention to avoid risk. People may simply conform to others’ behavior, through perceiving a type of social norm in observing others wearing masks (i.e., a descriptive norm; Cialdini et al., 1990; Lapinski and Rimal, 2005). During the 2009 H1N1 epidemic, wearing masks became a norm in Hong Kong (Lau et al., 2010). Ambiguous situations or states of anxiety – which are central characteristics of the present emergency – can also boost conformity (Taylor, 1953; Crutchfield, 1955). Wearing masks might relieve people’s anxiety regardless of masks’ realistic capacity to prevent infection. Another factor that may explain the decision to wear a mask is the affect heuristic, which predicts that our intuitive feelings toward activities or technologies define our perceptions of benefit as well as risk (Finucane et al., 2000; Slovic et al., 2002, 2004). Many people might wear masks simply because doing so promotes positive feelings, irrespective of masks’ objective effectiveness in reducing risks. Finally, a single-action bias in which people tend to adopt a single action against a risk may also be at play (Weber, 1997, 2006). The pandemic compels people to cope as well as they can, and wearing masks may be an accessible and convenient means to deal with the hardship. Our research examined how these six broad psychological reasons may explain the Japanese use of masks against COVID-19. Identifying influential psychological predictors can help us to improve our collective solutions.

## Materials and Methods

### Participants

We recruited participants through cross marketing, a leading market research company in Japan. Participants were recruited through electronic mail and accessed the designated website to participate in the survey. They earned small amounts of points for participating, with cash or a gift card awarded based on the number of accumulated points. We included only those who consented to participate in the study. There were 515 female participants and 485 male participants; 11.5% of female participants were in their 20s, 14.6% were in their 30s, 16.9% were in their 40s, 14.8% were in their 50s, 31.5% were in their 60s, 10.1% were in their 70s, and 0.8% were 80 years of age or older; 12.4% of male participants were in their 20s, 15.7% were in their 30s, 18.8% were in their 40s, 15.9% were in their 50s, 26.8% were in their 60s, 9.9% were in their 70s, and 0.6% were 80 years of age or older. The mean age of participants was 51.1 (*SD* = 15.5). The sample closely reflected the general population in Japan for sex, age, and residential area (the whole of Japan is divided into seven regions).

### Period

This survey was conducted between March 26 and 31, 2020. During this period, the total number of people infected with the 2019 novel coronavirus in Japan increased from 1,253 to 1,887, and the government announced that Japanese people should only go out if the trip was necessary or urgent.

### Procedure

Participants were asked about COVID-19 and the efficacy of masks, responding to six items using a five-point Likert scale (1 = *not at all* to 5 = *very much*). The items were the following:

- Perceived severity (severity): do you think that your disease condition would be serious if you had COVID-19?
- Perceived self-efficacy of wearing a mask for protection (protection): do you think that wearing a mask will keep you from being infected?
- Perceived efficacy of wearing a mask for preventing spread (prevention): do you think that people who have COVID-19 can avoid infecting others by wearing masks?
- Perceived norm to wear masks (norm): when you see other people wearing masks, do you think that you should wear a mask?
- Feeling relief when wearing masks (relief): do you think that you can ease your anxiety by wearing a mask?
- Impulse to take whatever actions are necessary (impulsion): do you think that you should “do whatever you can” to avoid COVID-19?

Participants were also asked about their frequency of wearing masks during this outbreak, using a three-point scale (1 = *I have not worn one at all*, 2 = *I have sometimes worn one*, and 3 = *I have usually worn one*).

## Results

**Figure 1:**
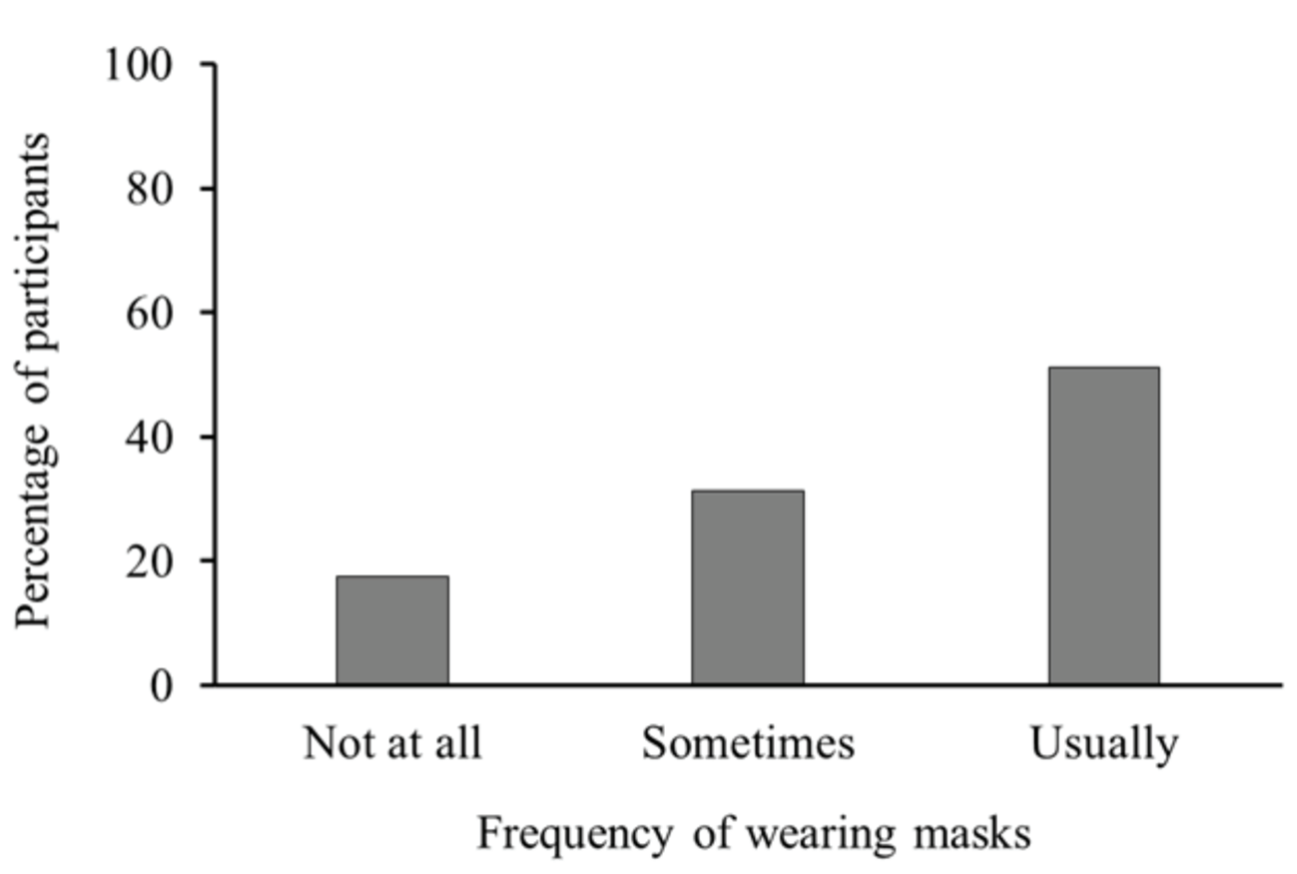
Percentage of participants based on frequency of using masks.

Figure 1 shows the results of participants’ mask usage, indicating that more than half usually wore masks from the beginning of the pandemic (Yamagata et al., 2020). Table 1 shows the descriptive statistics and correlations among variables regarding mask usage. We computed the product of severity and efficacy as an indicator of the effectiveness of wearing a mask (effectiveness). Reversed efficacy implies the inefficacy of wearing masks; thus, the product of severity and inefficacy is the perceived risk of infection under the mask-wearing condition (ineffectiveness). All psychological motivations were positively correlated to mask usage. Mask usage was regressed by the six psychological reasons to wear masks, removing the products above to avoid multicollinearity, and in order to compare the explanatory power of the psychological reasons. As indicated in Table 2, a powerful correlation was found between perception of norms and mask usage; conformity to the mask norm was the most influential determinant, given the standardized coefficient. Feeling relief from anxiety by wearing masks also promoted mask use. By contrast, frequency of mask usage depended much less on the participants’ perceived severity of the disease and the efficacy of masks in reducing infection risk both for themselves and for others. This implies that the perceived threat and risk reduction intentions were not the primary reason for wearing masks. Our analysis did not find a significant effect of willingness to take any action necessary. These six psychological factors explained one-third of the total variance in the frequency of wearing masks.

**Table 1:** Descriptive statistics and correlations among variables regarding mask usage.

S. No.	Measure	1	2	3	4	5	6	7	8	9
1.	Severity	-								
2.	Protection	0.22	-							
3.	Prevention	0.17	0.46	-						
4.	Impulsion	0.37	0.44	0.39	-					
5.	Norm	0.39	0.44	0.30	0.65	-				
6.	Relief	0.29	0.55	0.44	0.53	0.70	-			
7.	Effectiveness	0.74	0.77	0.38	0.48	0.48	0.50	-		
8.	Ineffectiveness	0.69	−0.50	−0.16	0.04	0.07	−0.12	0.01	-	
9.	Frequency of wearing mask	0.18	0.32	0.18	0.40	0.57	0.48	0.29	−0.04	-
	*M*	2.97	2.57	3.08	3.40	3.47	3.02	7.86	9.97	2.34
	*SD*	1.10	0.95	0.99	1.01	1.13	1.08	4.80	4.47	0.76
	*N* = 1,000									

**Table 2:** Results of multiple linear regression.

	Standardized coefficient
Severity	−0.06 (0.03)^*^
Protection	0.06 (0.03)^†^
Prevention	−0.06 (0.03)^*^
Impulsion	0.05 (0.04)
Norm	0.44 (0.04)^**^
Relief	0.16 (0.04)^**^

*Frequency of mask usage as explained by six variables. Standard errors are shown in brackets.*

*F*(6, 993) = 86.54, *R* ^2^ = 0.34.

† *p* < 0.10;

* *p* < 0.05;

** *p* < 0.01;

*N* = 1,000.

## Discussion

Even though the expectation of risk reduction (personal or collective) explained only small portion of mask usage, motivations superficially irrelevant to disease mitigation strongly promoted mask-wearing behavior; conformity to the social norm was the most prominent driving force for wearing masks. This tendency to conform was reported narratively during the H1N1 epidemic (Lau et al., 2010), but our research empirically confirmed the association. As mentioned in the context of the SARS pandemic, wearing masks can be a symbol of collective confrontation against a pandemic, even though its effectiveness in reducing personal risk remains uncertain (Syed et al., 2003). To establish effective strategies against COVID-19, social motivations such as conformity should be used to good advantage and embedded in nudge approaches. Nudges utilizing social norms are widely accepted and recommended by social scientists (Nyborg et al., 2016); therefore, we encourage policymakers to apply the effects of the social norm on the wearing of masks to promote collective efforts to combat COVID-19.

From the perspective of canonical models of risk-coping behavior, mitigation should be driven by intentions of risk reduction. However, our findings of the modest association between risk reduction expectations and behavior illustrate the complexities of risk-coping. Policymakers should also consider these complexities when conducting public relations. The positive correlation between behavior and relieving anxiety by wearing masks suggests that laypeople consider subjective feelings rather than objective risks. We did not examine whether this was derived from lack of knowledge, risk calculation ability, or human predisposition toward risks. However, this tendency should also be considered when delivering risk information.

This study has limitations, prompting recommendations for future research. Single items were used for measuring the constructs in the survey. Therefore, the measures may be associated with larger error variance compared with multiple scales. Furthermore, factors other than conformity, affect heuristic and single action bias were not included in the predictors of mask usage in the regression model. Despite these limitations, this study has empirically revealed that the expectation of risk reduction does not greatly promote mask-wearing countermeasures against COVID-19, suggesting that the nudge approach (i.e., taking advantage of people’s conformity) may be more promising. In future research, it will be necessary to construct more extensive models and design and conduct more elaborate surveys to comprehensively understand the public’s behaviors in relation to infection risks.

## Data Availability Statement

All datasets presented in this study are included in the article/Supplementary Material.

## Ethics Statement

The studies involving human participants were reviewed and approved by The Ethical Commission of the Faculty of Psychology at Doshisha University. Written informed consent for participation was not required for this study in accordance with the national legislation and the institutional requirements.

## Author Contributions

KN designed and performed the research. RY analyzed the data. TO and YS wrote and edited the paper. All authors contributed to the article and approved the submitted version.

### Conflict of Interest

The authors declare that the research was conducted in the absence of any commercial or financial relationships that could be construed as a potential conflict of interest.

## Supplementary Material

The dataset generated for this study can be found online at:

https://www.frontiersin.org/articles/10.3389/fpsyg.2020.01918/full#supplementary-material.
